# Rotational-progress-dependent pelvic, thoracic, and support-foot kinematics during a standardised Rumba spiral turn in highly trained adolescent female Latin dancers

**DOI:** 10.1371/journal.pone.0361699

**Published:** 2026-10-30

**Authors:** Mimi Guo, Muhamad Hafiz Bin Ismail, Diyana Zulaika Binti Abdul Ghani, Qi Liang, Yifei Gao

**Affiliations:** 1 Faculty of Educational Sciences and Technology, Universiti Teknologi Malaysia, Skudai, Johor, Malaysia; 2 College of Physical Education, Jiangsu Normal University, Xuzhou, Jiangsu, China; Universidade de Aveiro Escola Superior de Saude de Aveiro, PORTUGAL

## Abstract

Rumba turning involves continuous whole-body reorientation, but discrete kinematic summaries do not localise where consistent directional deviations occur across the turn. This exploratory study examined rotational-progress-dependent patterns of pelvic obliquity, thoracic frontal-plane angle, and right support-foot progression angle during the initial 360° Rumba spiral turn in highly trained adolescent female Latin dancers. Fifteen dancers aged 14–16 years completed five valid trials each (75 trials). Three-dimensional kinematics were recorded using a 10-camera Vicon system at 100 Hz. For each trial, accumulated pelvic rotation was used to define rotational progress, and the three outcome waveforms were resampled to 101 points across 0%–100% of the turn. The five trials from each participant were averaged point by point, yielding 15 participant-level representative waveforms per outcome. Two-tailed one-sample statistical parametric mapping (SPM{t}) tests identified intervals in which group mean waveforms differed from their corresponding Plug-in Gait model-defined 0° references, using a Bonferroni-adjusted primary α = 0.0167 across the three outcomes. Pelvic obliquity showed a positive deviation at 11.3%–47.2% and a negative deviation at 56.5%–97.3% of rotational progress. Thoracic frontal-plane angle showed a negative deviation at 13.2%–41.9% and positive deviations at 57.6%–83.0% and 85.7%–92.0%. Right support-foot progression angle showed negative, positive, and negative deviations at 0.0%–4.8%, 10.4%–49.1%, and 56.2%–100.0%, respectively. Sensitivity analyses without the additional across-outcome Bonferroni adjustment preserved the principal directional patterns, with only modest changes in cluster extent and continuity. These findings identify where, across rotational progress, the measured segmental orientations consistently departed from model-defined references during a standardised Rumba spiral turn, without implying normative targets of optimal dance technique.

## 1. Introduction

Turning is a fundamental component of Latin dance and requires continuous whole-body reorientation while maintaining prescribed postural and foot-placement characteristics throughout the movement. In Rumba, these demands are performed under task-specific conditions that include a relatively small support base, high-heeled footwear, and sustained changes in body orientation during rotation. Previous biomechanical studies of dance turning have reported differences in postural stability across turn phases and dancer experience levels [1], variations in trunk and whole-body kinematic demands according to the number of rotations performed [2], and continuously evolving supporting-limb and pelvis–trunk coordination during single-support pirouettes [3]. More recent work has also characterised phase-specific trunk and lower-limb kinematics during pirouettes performed by professional and experienced amateur dancers [4]. These observations support viewing turning as a continuously evolving kinematic task rather than as a single posture or endpoint event. Nevertheless, continuous descriptions of segmental orientation during standardised Rumba turning remain limited.

Previous biomechanical studies of dance turning have commonly used phase-specific or discrete kinematic summaries [1,2], while other work has analysed registered time-series or continuous kinematic trajectories [3,4]. Such approaches are useful for characterising selected aspects of performance, but discrete and phase-based summaries necessarily reduce continuous movement to a limited number of observations. Moreover, dancers may complete the same rotational task with differences in movement duration and local rotational velocity. For a movement defined explicitly by angular progression, expressing kinematic data relative to accumulated rotation offers a task-specific coordinate for locating directional deviations across the turn. One-dimensional statistical parametric mapping (SPM1d) enables statistical inference across an entire continuous waveform and can identify significant intervals without restricting the analysis to preselected discrete points [5–8]. Rotational-progress registration combined with continuous waveform inference therefore provides a framework for examining segmental orientation across a standardised turning task.

The pelvis, thorax, and support foot represent complementary aspects of body orientation during turning. Previous dance-turning studies have examined postural and trunk orientation [1,2,4], supporting-limb kinematics [3,4], and pelvis–trunk coordination [3], but these aspects have rarely been examined together as continuous functions of rotational progress during a standardised Rumba turn. A related study using the same participant cohort and standardised turning task evaluated repeated-trial waveform repeatability and measurement error across five repetitions [9]. That study addressed measurement consistency, whereas the present analysis addresses a different question: where, across rotational progress, do participant-averaged kinematic waveforms consistently depart from their model-defined reference orientations? The rotational-progress characteristics of pelvic obliquity, thoracic frontal-plane angle, and right support-foot progression angle during the initial Rumba spiral turn therefore remain to be established.

Accordingly, the present exploratory study examined pelvic obliquity, thoracic frontal-plane angle, and right support-foot progression angle as continuous waveforms during the initial 360° spiral turn in highly trained adolescent female Latin dancers. Individual trials were registered according to accumulated pelvic rotation, and participant-level waveforms were evaluated across the complete rotational-progress domain using one-dimensional SPM. The primary aim was to identify the rotational-progress intervals in which the group mean of each waveform differed from its corresponding Plug-in Gait model-defined 0° reference. These references were used as mathematical baselines for inference rather than as normative standards of optimal dance technique. It was hypothesised that the three kinematic waveforms would exhibit significant departures from their respective model-defined references over specific portions of rotational progress rather than remain centred on the reference throughout the entire primary rotational window.

## 2. Methods

### 2.1 Participants

This exploratory continuous-kinematic analysis included 15 highly trained adolescent female Latin dancers aged 14–16 years, each with five complete valid trials. Participants were recruited between 20 May and 20 June 2025, and data were collected between 5 and 10 July 2025.

For the purposes of this study, “highly trained” was operationalised as at least 5 years of systematic Latin dance training together with a top-six placing at a provincial-level or higher competition. Participants were excluded if they had sustained a lower-extremity, lumbopelvic, or trunk injury within the preceding 6 months that affected training or task performance; had a vestibular or balance disorder that could affect turning performance; or demonstrated an evident movement limitation during task familiarisation.

No formal a priori sample-size calculation was performed because the study was designed as an exploratory continuous-kinematic analysis. The analytical sample comprised all participants for whom five complete valid trials were available. The implications of the sample size for the precision and generalisability of the findings are considered in the Limitations. A related publication based on the same participant cohort and standardised turning task examined repeated-trial waveform repeatability and measurement error across five trials [9]. The present study addressed a distinct inferential question by averaging the five valid trials within each participant and testing the resulting participant-level waveforms against their respective model-defined 0° references using SPM1d.

The study was approved by the Research Ethics Committee of Universiti Teknologi Malaysia (UTMREC-2025–139) and was conducted in accordance with the Declaration of Helsinki [10]. Written informed consent was obtained from each participant’s legal guardian, and written assent was obtained from all participants before testing.

### 2.2 Standardised task and data collection

The experimental task was a standardised Rumba turning sequence comprising a 360° spiral turn, a 360° close-feet turn, and a 180° forward half-turn. The three components were performed consecutively as one continuous 900° sequence, without resetting between components. The initial 360° spiral turn constituted the primary analysis window. The subsequent close-feet turn and forward half-turn were completed to preserve the standardised task sequence but were not included in the primary analysis.

The structure of the standardised turning sequence and the location of the primary analysis window are illustrated in Fig 1.

**Fig 1:**
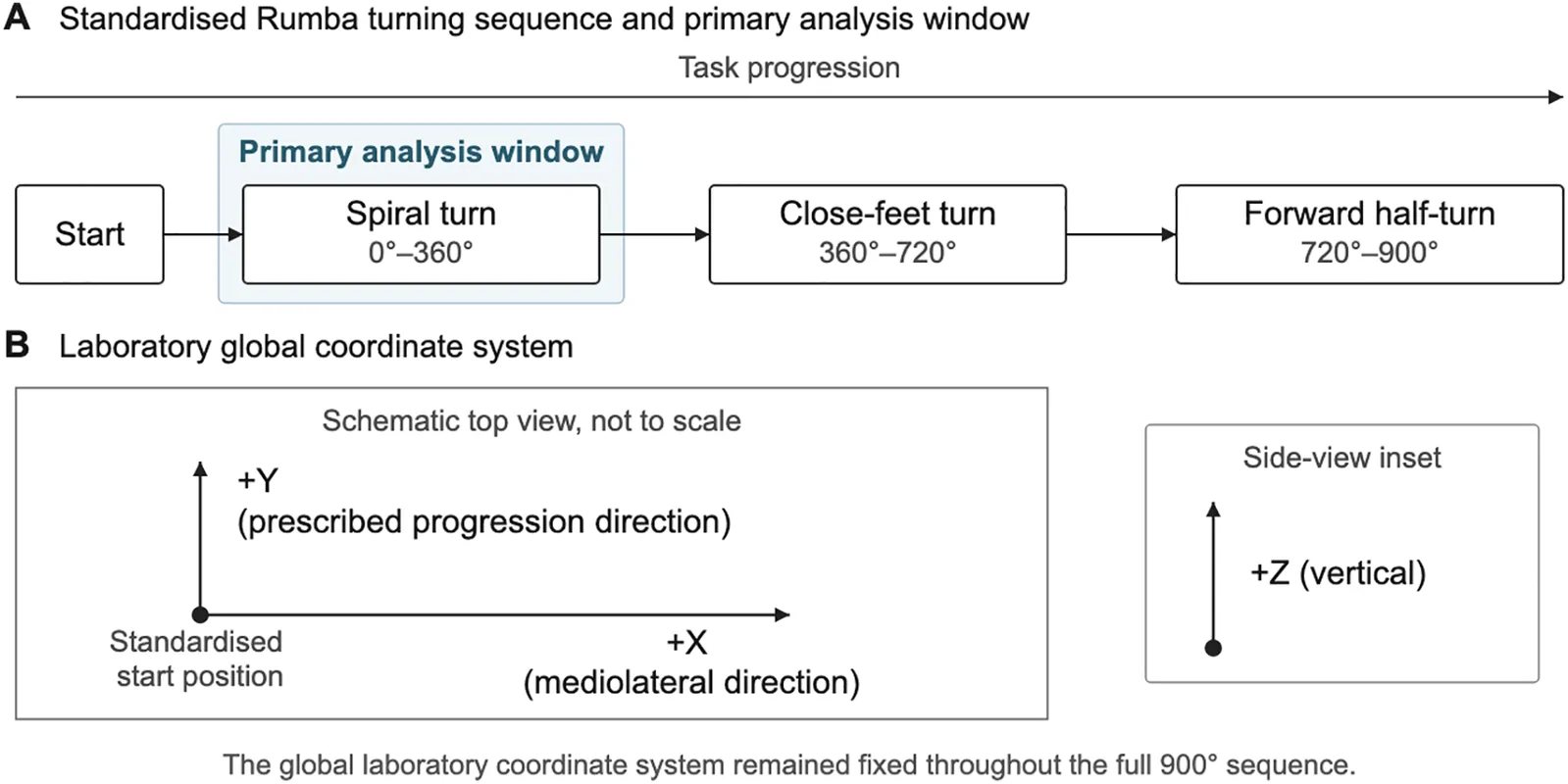
Standardised Rumba turning sequence, primary analysis window, and laboratory global coordinate system. (A) The standardised task comprised a 360° spiral turn, a 360° close-feet turn, and a 180° forward half-turn performed consecutively as one continuous 900° sequence. The initial 0°–360° spiral turn constituted the primary analysis window. (B) The laboratory global coordinate system was defined with the Y-axis aligned with the prescribed progression direction, the X-axis oriented mediolaterally and perpendicular to the progression line, and the Z-axis directed vertically upward. The global laboratory coordinate system remained fixed throughout the task. Angular outcome directions were interpreted according to the Plug-in Gait Progression Frame, as described in the Methods.

All participants began from the same standardised position, with body weight supported predominantly by the left leg and the right foot pointed forward in light contact with the floor. At movement initiation, the right foot was drawn inward before the dancer continued directly into the spiral turn. The right foot subsequently served as the support foot during the initial 360° spiral turn and was therefore used for the support-foot kinematic analysis. The sequence was performed to standardised Rumba music at 76 beats·min^−1^. All participants started from the same location and performed the task in the same prescribed direction. The laboratory global coordinate system, illustrated in Fig 1B, remained fixed throughout the task.

Three-dimensional kinematic data were collected on a flat laboratory floor using a 10-camera Vicon motion-capture system sampling at 100 Hz. Data were acquired using Vicon Nexus 2.6. Synchronised video recordings were retained for all trials to support verification of task execution and identification of the primary rotational window.

Before marker placement, the anthropometric measurements required to construct each participant-specific Plug-in Gait full-body model were obtained without footwear. Participants then wore standardised Latin dance shoes with a 5.5-cm heel. Reflective markers were attached according to the Plug-in Gait full-body marker set [11], followed by a static calibration trial within the capture volume. Marker placement, calibration, and initial data-quality checks were performed by the same trained laboratory team using a standardised procedure. Dynamic testing commenced only after marker identification and model construction had been verified.

Before the recorded trials, all participants completed a common preparatory routine lasting approximately 10–15 min. The routine comprised rhythmic joint-mobility exercises, including shoulder circumduction, trunk axial rotation, hip circumduction, knee flexion–extension, and ankle circumduction. Each exercise was typically performed for four eight-count musical phrases. These exercises were followed by basic Rumba steps and weight-transfer movements performed at low intensity.

Task familiarisation involved practising the complete 900° turning sequence, comprising the spiral turn, close-feet turn, and forward half-turn. Participants initially performed approximately five practice attempts without prescribed musical timing, followed by approximately five attempts to standardised Rumba music at 76 beats·min^−1^. The research team assessed tempo accuracy, movement sequence, and continuity, while marker visibility was checked to ensure adequate data capture. Familiarisation continued until each participant could complete the sequence without observable tempo errors, deviations from the prescribed movement sequence, movement interruptions, or marker obstruction.

Participants were tested sequentially, and the interval between the preparatory routine and recorded testing varied among participants. Some participants performed additional self-directed practice while awaiting testing. The number of these additional attempts was not systematically recorded. Familiarisation and additional practice attempts were excluded from the analysed trials.

Each participant completed five valid trials. Every attempt underwent immediate review of movement execution and data quality. Movement sequence and tempo accuracy were assessed by the research team, whereas marker visibility and trajectory completeness were reviewed by the laboratory engineer. Participants rested while the preceding attempt was reviewed, and the next attempt commenced only after the participant was ready.

Attempts involving evident tempo errors, incorrect movement sequences, movement interruptions, detachment of a key reflective marker, or substantial trajectory loss were classified as invalid and repeated during the same testing session. Data collection ceased once five valid trials had been obtained for each participant, resulting in 75 valid trials.

### 2.3 Signal preprocessing and quality control

Three-dimensional marker trajectories were reconstructed and labelled in Vicon Nexus 2.6. Marker gaps were addressed by an experienced laboratory engineer using rigid-body, pattern, or spline filling procedures selected according to marker location, segmental relationships, and gap characteristics. No fixed numerical gap-length threshold was used; gaps were filled only when the trajectory could be reconstructed reliably from the surrounding trajectory and available segmental relationships. All reconstructed and gap-filled trajectories were visually inspected before filtering and model calculation.

Marker trajectories were low-pass filtered in Vicon Nexus 2.6 using a fourth-order zero-phase Butterworth filter with a cutoff frequency of 10 Hz. The filtered trajectories were used to generate participant-specific Plug-in Gait model outputs, which were exported from Vicon Polygon 4.4 for subsequent analysis. No additional filtering was applied after export. Trials with key-marker detachment or trajectory loss that could not be reconstructed reliably were considered invalid.

### 2.4 Rotational-progress alignment and participant-level waveform construction

Rotational progress was defined from the third component of the Plug-in Gait pelvic-angle output (RPelvisAngles_Z), representing transverse-plane pelvic rotation about the vertical Z-axis [12], rather than from elapsed time. For each trial, the pelvic-rotation trace was unwrapped to remove angular discontinuities associated with rotational wrapping and to obtain a continuous angular trajectory. Rotation onset was defined as the point at which pelvic rotational velocity exceeded 30°·s^−1^ and remained above this threshold for at least 0.2 s. The identified onset was cross-checked against the synchronised video to confirm that it corresponded to the initial spiral turn rather than to preparatory movement before task initiation.

The unwrapped pelvic-rotation trace was direction-standardised so that progression in the prescribed turning direction was positive and was referenced to its value at rotation onset, such that accumulated rotational progress began at 0°. A cumulative-maximum transformation was then applied to the direction-standardised trace to generate a monotonically non-decreasing rotational-progress axis; consequently, local backward fluctuations produced plateaus in the registration axis rather than decreases in rotational progress. This transformation was applied only to the registration variable; the original model-derived outcome waveforms were not transformed to enforce monotonicity. The primary analysis window extended from rotation onset to the first point at which accumulated rotational progress reached or exceeded 360°.

Each valid trial was processed independently. The model-derived outcome waveforms within the initial 0°–360° window were resampled to 101 equally spaced rotational-progress points, representing 0%–100% of the primary rotation. The five valid trial waveforms from each participant were then averaged point by point to generate one participant-level representative waveform for each outcome variable. Consequently, the inferential analysis was based on 15 participant-level waveforms rather than treating the 75 trials as statistically independent observations.

### 2.5 Outcome variables and model-defined reference orientations

The primary outcomes were three model-derived angular waveforms obtained from the Plug-in Gait model [11]: pelvic obliquity (RPelvisAngles_Y), thoracic frontal-plane angle (RThoraxAngles_Y), and right support-foot progression angle (RFootProgressAngles_Z). These variables characterised the frontal-plane orientations of the pelvis and thorax and the transverse-plane orientation of the right support foot throughout the initial 360° spiral turn.

Pelvic obliquity represented the frontal-plane component of pelvic orientation relative to the Plug-in Gait Progression Frame. A value of 0° represented zero model-defined pelvic obliquity. Positive values indicated upward obliquity of the right side of the pelvis, whereas negative values indicated the opposite orientation [12].

Thoracic frontal-plane angle represented the frontal-plane component of thoracic orientation relative to the Plug-in Gait Progression Frame. A value of 0° represented zero model-defined thoracic frontal-plane inclination. Positive values indicated leftward thoracic inclination, whereas negative values indicated rightward inclination [13].

Right support-foot progression angle represented the transverse-plane orientation of the right support foot relative to the Plug-in Gait Progression Frame. A value of 0° represented zero transverse-plane rotation relative to the model-defined progression direction. Positive values indicated internal rotation of the right support foot relative to this reference, whereas negative values indicated external rotation [12].

All three outcomes were retained as continuous 101-point waveforms throughout the primary rotational window. No discrete peak, trough, timing, or total-excursion variables were used for the primary inferential analysis. The model-defined 0° orientations were used solely as mathematical references for statistical inference. Accordingly, a significant deviation from 0° was interpreted as a consistent directional departure from the corresponding model reference and not as evidence of an optimal, suboptimal, or technically incorrect dance posture.

The angle components were interpreted according to the Plug-in Gait output convention and its Progression Frame rather than directly from the laboratory axis labels described above [12,13].

### 2.6 Statistical analysis

All inferential analyses were conducted at the participant level. For each of the three primary kinematic outcomes, the five valid trial waveforms were averaged point by point within each participant, yielding one 101-point representative waveform per participant and outcome. Accordingly, the inferential sample comprised 15 participant-level waveforms for each outcome. Group mean waveforms and pointwise standard deviations were calculated for descriptive presentation.

Normality of the participant-level residuals was assessed pointwise across the 101 rotational-progress nodes using Shapiro–Wilk tests, with Holm correction applied across nodes within each outcome [14]. No significant departures from normality were detected for any of the three primary outcomes.

One-dimensional statistical parametric mapping (SPM1d) was used to identify rotational-progress intervals in which the group mean of the participant-level waveforms differed significantly from the corresponding model-defined 0° reference [5,6]. Separate two-tailed one-sample SPM{t} tests were performed for pelvic obliquity, thoracic frontal-plane angle, and right support-foot progression angle across the complete 101-point rotational-progress domain. For each outcome, the null hypothesis was that the population mean waveform was equal to 0° throughout the primary rotational window.

SPM1d controlled the family-wise error rate across the continuous rotational-progress domain within each outcome test. To additionally control multiplicity across the three primary outcomes, a Bonferroni adjustment was applied, giving a primary significance level of α = 0.05/3 = 0.0167. For each significant supra-threshold cluster, the rotational-progress boundaries, cluster-level p value, signed extremum SPM{t} statistic, and rotational-progress location of the extremum were retained for reporting. Positive and negative clusters were classified according to the sign of the SPM{t} field, which corresponded to the direction of the group mean deviation from the model-defined 0° reference.

To assess whether the principal findings depended materially on the additional across-outcome Bonferroni adjustment, a sensitivity analysis repeated the three two-tailed one-sample SPM{t} tests using a test-wise α = 0.05 without the additional across-outcome correction. This analysis was used to evaluate the robustness of the location and directional pattern of the significant intervals to the multiplicity-control strategy and did not replace the Bonferroni-adjusted primary inference.

SPM analyses were performed in Python using the spm1d package (version 0.4.53) [6]. Reproducibility Python code implementing the reported data-processing and statistical-analysis pipeline is provided as S1 Code.

## 3. Results

### 3.1 Participant characteristics and valid trials in the primary analysis window

Participant characteristics are presented in Table 1. Each participant contributed five complete valid trials, resulting in a total of 75 valid trials for continuous waveform analysis within the primary 360° rotational window.

**Table 1:** Characteristics of the study participants (n = 15).

Characteristic	Mean ± SD
Age (years)	14.93 ± 0.80
Height (cm)	163.87 ± 2.77
Body mass (kg)	52.79 ± 3.58
Body mass index (kg·m^−2^)	19.69 ± 1.66
Latin dance training experience (years)	6.47 ± 1.30

The retained trials were resampled along the rotational-progress axis, and the five trial waveforms from each participant were averaged point by point to generate one representative waveform per participant. Therefore, all inferential analyses were conducted using 15 participant-level representative waveforms.

### 3.2 Pelvic obliquity across rotational progress

Pelvic obliquity exhibited a biphasic waveform pattern across the primary rotational window (Table 2 and Fig 2). The waveform initially showed a positive deviation from the model-defined 0° reference and subsequently demonstrated a negative deviation during the latter portion of the rotation.

**Table 2:** Primary one-sample SPM{t} results across the initial 360° rotational window.

Variable	Direction of deviation	Significant interval (%RP)	Cluster-level p	Signed extremum SPM{t} statistic	Extremum location (%RP)
Pelvic obliquity	Positive—right side upward	11.3–47.2	<0.001	11.42	27
Pelvic obliquity	Negative—right side downward	56.5–97.3	<0.001	−11.13	88
Thoracic frontal-plane angle	Negative—rightward inclination	13.2–41.9	<0.001	−10.77	23
Thoracic frontal-plane angle	Positive—leftward inclination	57.6–83.0	<0.001	5.94	67
Thoracic frontal-plane angle	Positive—leftward inclination	85.7–92.0	0.005	5.53	89
Right support-foot progression angle	Negative—external rotation	0.0–4.8	0.005	−10.81	0
Right support-foot progression angle	Positive—internal rotation	10.4–49.1	<0.001	34.12	33
Right support-foot progression angle	Negative—external rotation	56.2–100.0	<0.001	−35.14	77

RP, rotational progress. For pelvic obliquity, positive values indicate upward obliquity of the right side of the pelvis and negative values indicate right-side downward obliquity. For thoracic frontal-plane angle, positive values indicate leftward inclination and negative values indicate rightward inclination. For right support-foot progression angle, positive values indicate internal rotation and negative values indicate external rotation relative to the Plug-in Gait Progression Frame. Significant clusters were identified using two-tailed one-sample SPM{t} tests with the Bonferroni-adjusted primary significance level of α = 0.0167. All one-sample SPM{t} tests had 14 degrees of freedom.

**Fig 2:**
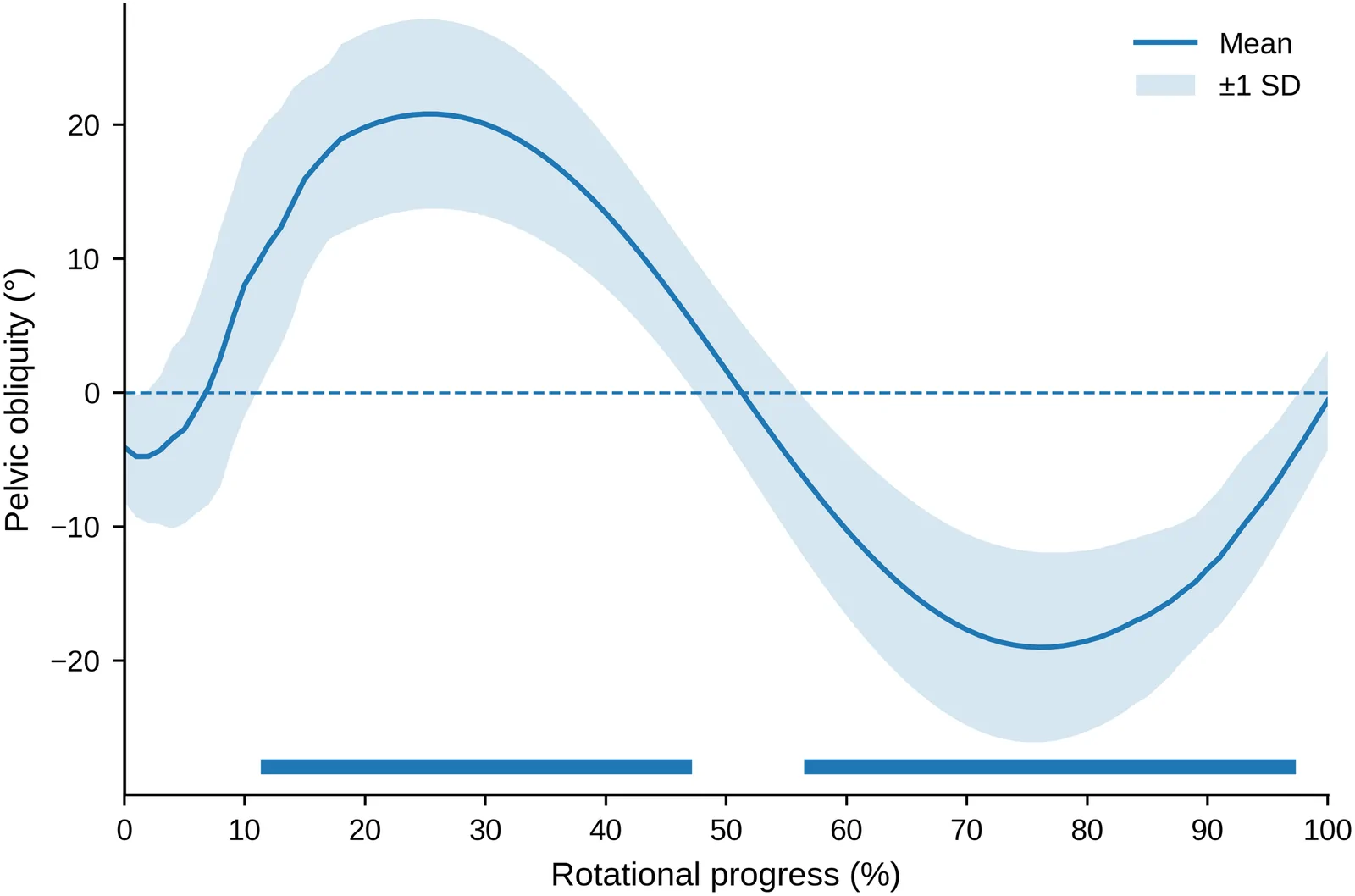
Pelvic obliquity across the primary rotational window. The solid line represents the group mean waveform and the shaded band represents ±1 SD across the 15 participant-level representative waveforms. The dashed horizontal line indicates the model-defined 0° reference. Thick horizontal bars indicate rotational-progress intervals in which pelvic obliquity differed significantly from the model-defined reference in the primary two-tailed one-sample SPM{t} analysis using the Bonferroni-adjusted significance level (α = 0.0167).

One-sample SPM{t} analysis identified two significant rotational-progress intervals in which pelvic obliquity differed from the model-defined reference: 11.3–47.2% and 56.5–97.3% of rotational progress (Table 2). The first interval represented a positive deviation, whereas the second interval represented a negative deviation according to the Plug-in Gait output convention.

### 3.3 Thoracic frontal-plane angle across rotational progress

Thoracic frontal-plane angle showed a negative deviation from the model-defined 0° reference during the earlier portion of rotational progress and positive deviations during the later portion (Table 2 and Fig 3).

**Fig 3:**
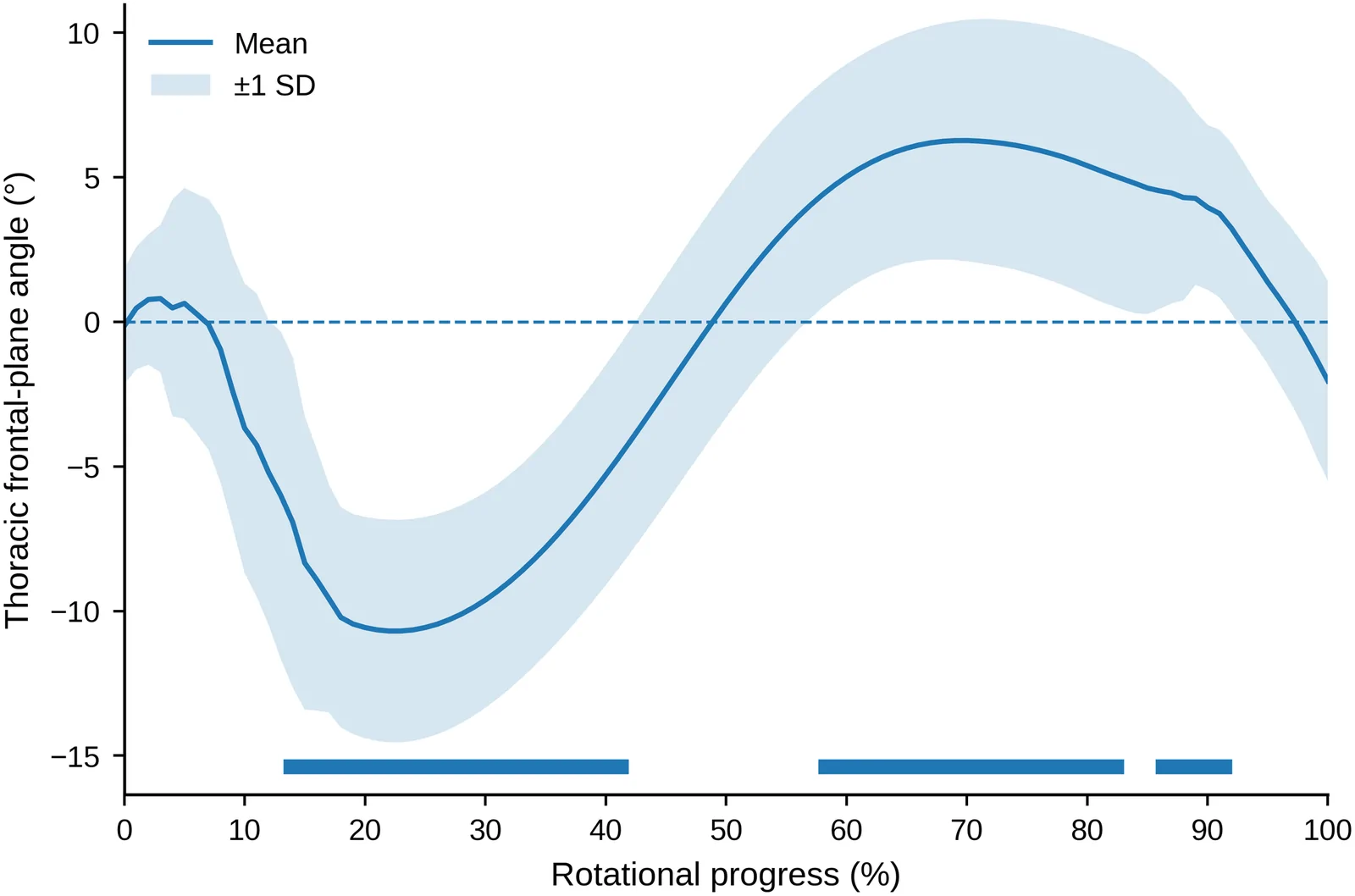
Thoracic frontal-plane angle across the primary rotational window. The solid line represents the group mean waveform and the shaded band represents ±1 SD across the 15 participant-level representative waveforms. The dashed horizontal line indicates the model-defined 0° reference. Thick horizontal bars indicate rotational-progress intervals in which thoracic frontal-plane angle differed significantly from the model-defined reference in the primary two-tailed one-sample SPM{t} analysis using the Bonferroni-adjusted significance level (α = 0.0167).

One-sample SPM{t} analysis identified three significant rotational-progress intervals in which thoracic frontal-plane angle differed from the model-defined reference: 13.2–41.9%, 57.6–83.0%, and 85.7–92.0% of rotational progress (Table 2). The first interval represented a negative deviation, whereas the latter two intervals represented positive deviations according to the Plug-in Gait output convention.

### 3.4 Right support-foot progression angle across rotational progress

Right support-foot progression angle showed a sequential negative–positive–negative deviation pattern across the primary rotational window (Table 2 and Fig 4).

**Fig 4:**
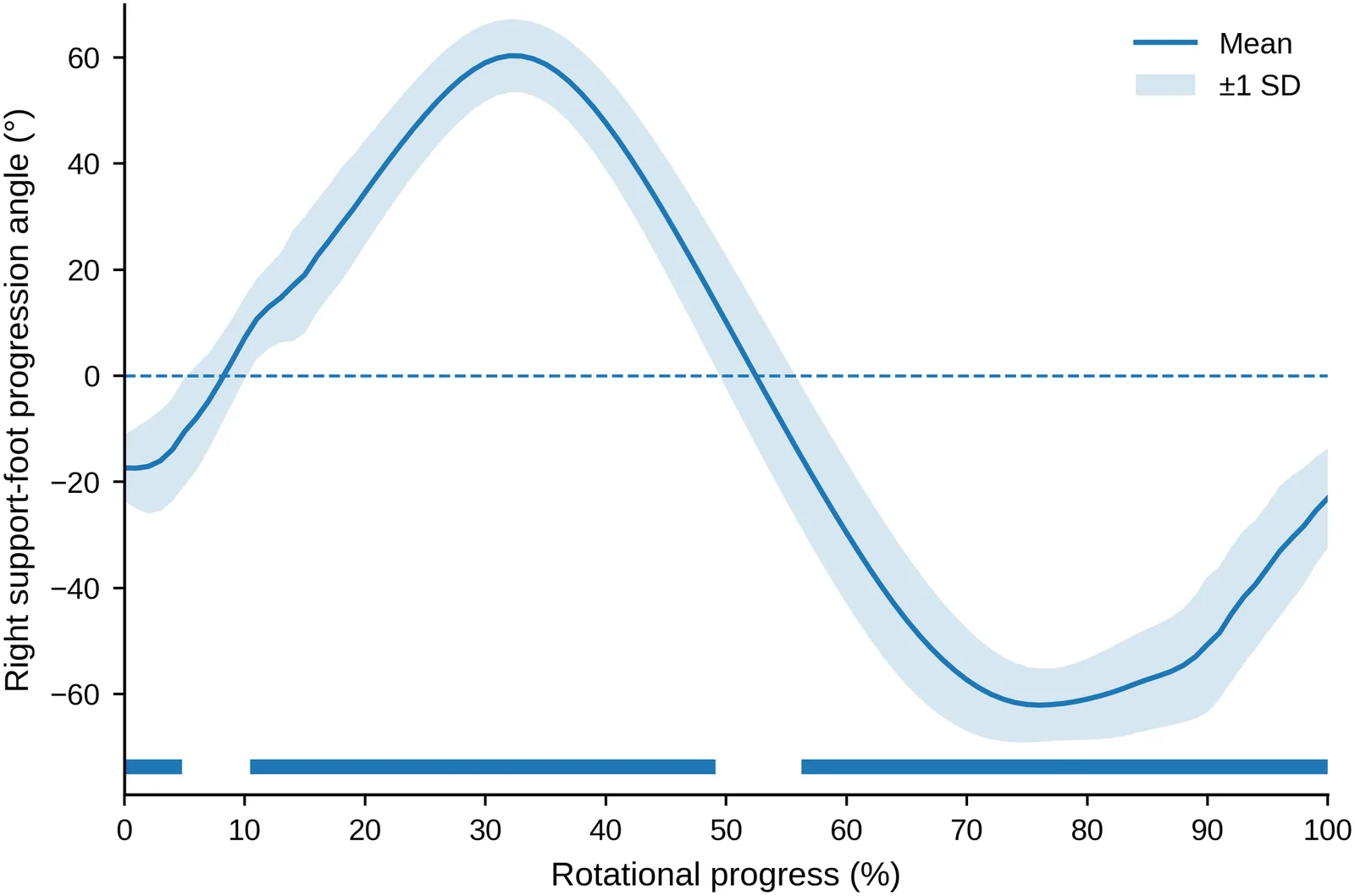
Right support-foot progression angle across the primary rotational window. The solid line represents the group mean waveform and the shaded band represents ±1 SD across the 15 participant-level representative waveforms. The dashed horizontal line indicates the model-defined 0° reference. Thick horizontal bars indicate rotational-progress intervals in which right support-foot progression angle differed significantly from the model-defined reference in the primary two-tailed one-sample SPM{t} analysis using the Bonferroni-adjusted significance level (α = 0.0167).

One-sample SPM{t} analysis identified three significant rotational-progress intervals in which right support-foot progression angle differed from the model-defined reference: 0.0–4.8%, 10.4–49.1%, and 56.2–100.0% of rotational progress (Table 2). According to the Plug-in Gait output convention, these intervals represented external rotation, internal rotation, and external rotation of the right support foot, respectively.

### 3.5 Sensitivity to the across-outcome multiplicity adjustment

Repeating the three two-tailed one-sample SPM{t} tests at a test-wise α = 0.05 without the additional across-outcome Bonferroni adjustment preserved the overall directional pattern identified in the primary analysis, although some cluster boundaries and continuity changed.

For pelvic obliquity, an additional short negative interval was identified at 0.0–2.3% of rotational progress, while the principal positive and negative intervals extended from 10.5% to 47.8% and from 55.5% to 97.9%, respectively. For thoracic frontal-plane angle, the negative interval extended from 11.7% to 42.9%, whereas the two positive intervals identified separately in the primary analysis became a single continuous positive interval from 56.0% to 92.8%. For right support-foot progression angle, the same sequence of negative, positive, and negative deviations was retained at 0.0–5.3%, 10.1–49.6%, and 55.7–100.0%, respectively.

Overall, removal of the additional across-outcome Bonferroni adjustment altered the extent and, for the thoracic outcome, the continuity of some significant clusters, but did not materially change the principal inferential pattern.

## 4. Discussion

Continuous descriptions of segmental orientation during standardised Rumba turning remain limited, particularly when movement is registered to rotational progress rather than elapsed time. This limits identification of where within the turn consistent pelvic, thoracic, and support-foot directional deviations occur. The present study therefore used rotational-progress-aligned waveform inference to localise these deviations during the initial 360° spiral turn in highly trained adolescent female Latin dancers. The analysis was intended to provide a task-specific descriptive account of segmental orientation rather than to establish causal control mechanisms or normative technical targets.

### 4.1 Principal findings and interpretive context

This exploratory study used participant-averaged, rotational-progress-aligned continuous kinematic analysis to characterise pelvic obliquity, thoracic frontal-plane angle, and right support-foot progression angle during the initial 360° spiral turn in highly trained adolescent female Latin dancers. Relative to their model-defined 0° references, pelvic obliquity showed a positive deviation during an earlier interval and a negative deviation during a later interval; thoracic frontal-plane angle showed an earlier negative deviation and later positive deviations; and right support-foot progression angle followed a negative–positive–negative directional pattern. These principal patterns were retained in the sensitivity analysis without the additional across-outcome Bonferroni adjustment, although the boundaries and continuity of some significant intervals changed.

These findings represent group-level directional departures from the Plug-in Gait model-defined reference orientations. The 0° values were mathematical model references rather than normative or optimal dance positions; accordingly, significant intervals identify where the measured orientations consistently departed from those references, not whether a posture was technically correct or incorrect. The findings should therefore be interpreted as rotational-progress-dependent kinematic characteristics of the standardised turn rather than as evidence of superior movement strategies or causal control mechanisms.

### 4.2 Pelvic obliquity across rotational progress

Pelvic obliquity showed a positive deviation from the model-defined 0° reference during much of the earlier portion of rotational progress and a negative deviation during much of the later portion. The principal pelvic finding was therefore the presence of opposite directional deviations across different portions of the initial 360° spiral turn, rather than a sustained deviation in one direction throughout the movement.

Previous biomechanical research has shown that pelvic orientation and its relationship with the trunk vary according to the movement task. During walking turns, trunk–pelvis coordination has been shown to differ across portions of the turning movement [15], while continuous pelvis–trunk and supporting-limb coordination has also been described during single-support ballet pirouettes [3]. Latin-dance-specific studies have provided complementary evidence: professional Latin dancers demonstrated characteristic pelvic tilt and spinal postures under different standing, footwear, and dance conditions [16], and dance-specific Latin postures have been associated with differences in pelvic torsion and other upper-body measures [17]. These studies examined different planes, tasks, and outcome definitions from those used here and therefore should not be interpreted as direct equivalents of the present pelvic-obliquity waveform. The present analysis extends this literature by localising positive and negative frontal-plane pelvic deviations according to accumulated rotational progress during a standardised Rumba spiral turn. These findings describe the distribution of pelvic orientation across the turn but do not establish active correction, compensation, or a specific stabilising function.

### 4.3 Thoracic frontal-plane angle across rotational progress

Thoracic frontal-plane angle showed a negative deviation from the model-defined 0° reference during the earlier portion of rotational progress and positive deviations during the later portion. The principal thoracic finding was therefore the occurrence of opposite directional deviations across earlier and later portions of the initial 360° spiral turn.

Previous dance-biomechanics studies have provided evidence of task-dependent trunk organisation. Dancers and non-dancers have been shown to differ in aspects of trunk and lower-limb intersegmental coordination during repeated dance movements [18], while trunk-related physical characteristics have been associated with static and dynamic postural-control measures in Latin dancers [19]. In Latin dance specifically, upper-body posture has also been shown to differ across dance-specific positions [17]. During ballet turning, changes in preparatory stance configuration have been shown to influence the inclination of the torso rotation axis and supporting-foot displacement [20], complementing earlier evidence of phase-dependent trunk kinematics during pirouettes [1,4]. Although these studies used different tasks and outcome measures, together they have shown that trunk orientation and coordination vary across dance movements. The present findings extend this perspective by localising intervals of negative and positive thoracic frontal-plane deviation across rotational progress during a standardised Rumba spiral turn. The observed pattern should be interpreted as a descriptive characteristic of thoracic orientation across rotational progress rather than as evidence of compensatory or stabilising interactions with the pelvis or other body segments.

### 4.4 Right support-foot progression angle across rotational progress

Right support-foot progression angle showed a sequential negative–positive–negative deviation pattern across the primary rotational window. A short negative deviation occurred at the beginning of rotational progress, followed by a positive deviation through much of the earlier-to-middle portion of the turn and a subsequent negative deviation across most of the later portion. According to the Plug-in Gait output convention, these intervals corresponded to external, internal, and external orientation of the right support foot relative to the model-defined progression reference.

Previous dance-biomechanics research has suggested that the organisation of the supporting limbs is sensitive to rotational and technical task demands. Skilled dancers were found to modify the relative contributions of the two legs when progressing from single to double pirouettes [21], while differences in supporting-leg joint ranges of motion were reported between classical and jazz pirouettes [22]. More directly relevant to foot orientation, kinematic studies of ballet turnout have shown that total lower-limb rotation is distributed across the hip, knee, and more distal segments [23], that foot progression angle can vary between dance positions and limb settings [24], and that turnout assessment cannot be represented solely by hip external rotation because non-hip contributions are substantial [25]. A recent dynamic analysis further demonstrated that hip, knee, and ankle external-rotation contributions differed across movement phases and dance tasks [26].

Previous studies of Latin dancers have also shown that lower-limb kinematics vary according to performer and task conditions. Latin dancers have shown stance-phase differences in ankle and hip rotation compared with non-dancers during walking [27], together with distinct gait and balance characteristics [28]. During Jive movements, forefoot and entire-foot support strategies have been associated with different ankle-angle and ankle-velocity profiles [29], while a Cha-Cha training study reported changes in lower-limb joint angles together with dance-specific technical-quality measures [30]. These findings do not define an optimal support-foot angle for Rumba turning, but they reinforce the importance of treating foot and lower-limb orientation as movement-specific rather than as a fixed configuration. The present findings add a continuous description of this feature during a standardised Rumba spiral turn by localising intervals of external and internal support-foot orientation relative to the model-defined progression reference. These observations characterise the orientation of the support foot relative to the progression reference and do not establish an optimal foot position or a specific mechanical contribution to turning performance.

### 4.5 Methodological robustness and interpretive implications

Continuous one-dimensional inference is designed to retain the structure of biomechanical waveforms rather than reducing them entirely to isolated scalar observations [5–8], and validation work has supported SPM inference for one-dimensional biomechanical data under non-uniform and heterogeneous smoothness conditions [31]. Nevertheless, registration remains an important consideration in trajectory-level analysis because differences in event timing and waveform alignment can influence continuous statistical outcomes [32]. SPM also represents only one approach within the broader family of continuum-data methods: Bayesian extensions of SPM have been described [33], and continuous biomechanical responses have been combined with alternative re-parameterisation strategies such as arc-length registration [34]. Functional-data approaches have also provided additional frameworks for analysing movement curves [35]. Against this methodological background, expressing the present kinematic waveforms relative to accumulated rotational progress provided a task-specific registration axis for locating directional deviations within the initial 360° spiral turn. Combined with continuous SPM analysis, this framework preserved the continuous structure of each waveform and allowed significant intervals to be expressed directly in relation to rotational progress rather than reducing each outcome to a small number of discrete angular measures. This approach was used to characterise the present task and was not designed to establish superiority over alternative normalisation or functional-data procedures.

The primary analyses applied a Bonferroni-adjusted significance level across the three outcomes in addition to family-wise error control across the continuous domain within each SPM test. This provided conservative protection against multiplicity across outcomes, although such an adjustment may reduce sensitivity to shorter or weaker departures from the reference. When the analyses were repeated at a test-wise α = 0.05 without the additional across-outcome adjustment, the principal directional patterns remained unchanged. The main differences involved modest shifts in cluster boundaries and the merging of the two later thoracic positive clusters into a single continuous interval. These findings indicate that the main inferential pattern was qualitatively consistent across the two multiplicity-control approaches, whereas the exact extent and continuity of individual significant intervals depended on the significance threshold. Accordingly, the continuous intervals are most appropriately viewed as descriptive locations of consistent group-level deviation within the standardised task rather than normative technical targets.

### 4.6 Limitations

The present findings should be interpreted within the population and task examined. The relatively small and homogeneous sample comprised 15 highly trained adolescent female Latin dancers, which limits the precision and generalisability of the findings beyond this specialised population. The identified patterns should therefore be extrapolated cautiously to dancers of different ages, sexes, training levels, or dance backgrounds. In addition, the analysis was confined to the initial 360° spiral turn of the standardised Rumba sequence, and the findings should not be assumed to represent other components of the sequence or other turning tasks. Because the support-foot analysis was restricted to the right foot during the prescribed spiral turn, these findings should not be generalised directly to turns performed with the opposite support limb or turning direction.

The analysis focused on continuous kinematic waveforms of the pelvis, thorax, and right support foot. Consequently, the present results characterise segmental orientation across rotational progress but do not determine the kinetic or neuromuscular mechanisms underlying these patterns. In addition, the Plug-in Gait 0° orientations were model-defined mathematical references rather than normative standards of dance technique, which limits interpretation of the observed deviations as technically desirable or undesirable positions. Future studies involving broader dancer populations and additional turning tasks may determine how consistently these rotational-progress-specific patterns are expressed under different performer and task conditions.

## 5. Conclusion

In this exploratory sample of highly trained adolescent female Latin dancers, continuous rotational-progress analysis of the initial 360° Rumba spiral turn identified distinct rotational-progress-dependent directional deviation patterns in pelvic obliquity, thoracic frontal-plane angle, and right support-foot progression angle relative to their model-defined 0° references. These principal directional patterns were retained in the sensitivity analysis, indicating qualitative consistency of the main findings across the two multiplicity thresholds examined. Together, the results provide a continuous kinematic description of where segmental orientations consistently departed from their model-defined references during the turn. These deviations, however, should not be interpreted as normative targets of optimal dance technique. These findings are limited to a small, homogeneous sample of adolescent female dancers and to kinematic data from the initial right-support 360° spiral turn; they should not be extrapolated directly to other dancer populations, opposite-support turning conditions, or underlying kinetic or neuromuscular mechanisms.

## Supporting information

S1 Data Anonymised processed data underlying the reported analyses. The workbook contains the 75 processed trial-level rotational-progress-aligned waveforms, the 15 participant-level representative waveforms used for inference, source data underlying the reported figures, exact primary and sensitivity SPM results, nodewise normality results, an anonymised processing audit, validation results, and a data dictionary.
(XLSX)

S1 Code Reproducibility Python code implementing the rotational-progress alignment and statistical-analysis pipeline. The archive contains scripts documenting the raw-to-waveform registration procedure, construction of participant-level representative waveforms, nodewise Shapiro–Wilk screening with Holm adjustment, primary and sensitivity one-sample SPM{t} analyses, and generation of the principal waveform figures, together with software requirements and validation files.
(ZIP)

## References

[1] Lin C-W, Su F-C, Lin C-F. Kinematic analysis of postural stability during ballet turns (pirouettes) in experienced and novice dancers. Front Bioeng Biotechnol. 2019;7:290. doi: 10.3389/fbioe.2019.00290 31709249 PMC6823550

[2] Kim J, Wilson MA, Singhal K, Gamblin S, Suh C-Y, Kwon Y-H. Generation of vertical angular momentum in single, double, and triple-turn pirouette en dehors in ballet. Sports Biomech. 2014;13(3):215–29. doi: 10.1080/14763141.2014.933580 25325768

[3] Lott MB, Xu G. Joint angle coordination strategies during whole body rotations on a single lower-limb support: An investigation through ballet pirouettes. J Appl Biomech. 2020;36(2):103–12. doi: 10.1123/jab.2019-0209 32126523

[4] Tsubaki Y, Kawano Y, Lin C-F, Kuno-Mizumura M. Laterality in body coordination of professional and amateur ballet dancers while performing a single pirouette with pointe shoes. Med Probl Perform Artists. 2024;39(1):8–17. doi: 10.21091/mppa.2024.1002 38413826

[5] Pataky TC. Generalized n-dimensional biomechanical field analysis using statistical parametric mapping. J Biomech. 2010;43(10):1976–82. doi: 10.1016/j.jbiomech.2010.03.008 20434726

[6] Pataky TC. One-dimensional statistical parametric mapping in Python. Comput Methods Biomech Biomed Eng. 2012;15(3):295–301. doi: 10.1080/10255842.2010.527837 21756121

[7] Pataky TC, Vanrenterghem J, Robinson MA. Zero- vs. one-dimensional, parametric vs. non-parametric, and confidence interval vs. hypothesis testing procedures in one-dimensional biomechanical trajectory analysis. J Biomech. 2015;48(7):1277–85. doi: 10.1016/j.jbiomech.2015.02.051 25817475

[8] Yona T, Kamel N, Cohen-Eick G, Ovadia I, Fischer A. One-dimension statistical parametric mapping in lower limb biomechanical analysis: A systematic scoping review. Gait Posture. 2024;109:133–46. doi: 10.1016/j.gaitpost.2024.01.018 38306782

[9] Guo M, Ismail MHB, Abdul Ghani DZB, Liang Q, Gao Y. Trial-to-trial repeatability and curve-level reliability of rotational-progress-aligned kinematic waveforms during the primary 360° rotation of a rumba turning sequence. J Appl Biomech. 2026:1–8. doi: 10.1123/jab.2026-0127 42632627

[10] World Medical Association. World Medical Association Declaration of Helsinki: Ethical principles for medical research involving human participants. JAMA. 2025;333:71–4. doi: 10.1001/jama.2024.21972 39425955

[11] Vicon Motion Systems Ltd. Full body modeling with Plug-in Gait—Nexus 2.6 documentation [Internet]. Oxford: Vicon Motion Systems Ltd.; [cited 2026 Sept 6]. Available from: https://docs.vicon.com/display/Nexus26/Full+body+modeling+with+Plug-in+Gait#FullbodymodelingwithPlug-inGait-MarkersetsforPlug-inGaitfullbodymodeling

[12] Vicon Motion Systems Ltd. What are the lower body segment angles from Plug-in Gait? [Internet]. Oxford: Vicon Motion Systems Ltd. [cited 2026 Sept 6]. Available from: https://www.vicon.com/support/faqs/what-are-the-lower-body-segment-angles-from-plug-in-gait/

[13] Vicon Motion Systems Ltd. What are the upper body segment angles from Plug-in Gait? [Internet]. Oxford: Vicon Motion Systems Ltd.; [cited 2026 Sept 6]. Available from: https://www.vicon.com/support/faqs/what-are-the-upper-body-segment-angles-from-plug-in-gait/

[14] Holm S. A simple sequentially rejective multiple test procedure. Scand J Stat. 1979;6:65–70. doi: 10.2307/4615733

[15] Smith JA, Kulig K. Trunk-pelvis coordination during turning: A cross sectional study of young adults with and without a history of low back pain. Clin Biomech (Bristol). 2016;36:58–64. doi: 10.1016/j.clinbiomech.2016.05.011 27214247

[16] Muyor JM, Zemková E, Chren M. Effects of Latin style professional dance on the spinal posture and pelvic tilt. J Back Musculoskelet Rehabil. 2017;30(4):791–800. doi: 10.3233/BMR-150448 28372311

[17] Wanke EM, Mörl-Kreitschmann M, Holzgreve F, Groneberg D, Ohlendorf D. Upper body posture in Latin American dancers: A quantitative cross-sectional study comparing different postures. BMC Sports Sci Med Rehabil. 2023;15(1):66. doi: 10.1186/s13102-023-00672-w 37098589 PMC10131306

[18] Jarvis DN, Smith JA, Kulig K. Trunk coordination in dancers and nondancers. J Appl Biomech. 2014;30(4):547–54. doi: 10.1123/jab.2013-0329 24979816

[19] Özkal Ö, Demircioğlu A, Topuz S. Clarifying the relationships between trunk muscle endurance, respiratory muscle strength and static/dynamic postural control in Latin dancers. Sports Biomech. 2025;24(6):1562–75. doi: 10.1080/14763141.2024.2301984 38193516

[20] Lin C-W, Wu H-W, Su F-C, Lin C-F. Influence of stance configuration on postural control while performing ballet turns (pirouette) in novices and experienced dancers. Med Probl Perform Artists. 2025;40(1):11–9. doi: 10.21091/mppa.2025.01002 40111103

[21] Zaferiou AM, Wilcox RR, McNitt-Gray JL. Modification of impulse generation during pirouette turns with increased rotational demands. J Appl Biomech. 2016;32(5):425–32. doi: 10.1123/jab.2015-0314 27046934

[22] Błażkiewicz M. Joint loads and muscle force distribution during classical and jazz pirouettes. Acta Bioeng Biomech. 2021;23(1):3–13. doi: 10.37190/abb-01675-2020-02 34846018

[23] Quanbeck AE, Russell JA, Handley SC, Quanbeck DS. Kinematic analysis of hip and knee rotation and other contributors to ballet turnout. J Sports Sci. 2017;35(4):331–8. doi: 10.1080/02640414.2016.1164335 27015006

[24] Gorwa J, Kabaciński J, Murawa M, Fryzowicz A. On the track of the ideal turnout: Electromyographic and kinematic analysis of the five classical ballet positions. PLoS One. 2020;15(3):e0230654. doi: 10.1371/journal.pone.0230654 32210478 PMC7094876

[25] Angioi M, Hodgson K, Okholm Kryger K. An updated systematic review of turnout position assessment protocols used in dance medicine and science research. J Dance Med Sci. 2021;25(1):55–71. doi: 10.12678/1089-313X.031521h 33706856

[26] Manfrim LC, Orselli MIV, Portela BM, Moutinho MO, Caravaggi P, Sacco ICN. Lower limb joints’ contributions to ballet turnout during unipodal and bipodal jumps in fifth position in pre-professional dancers. PeerJ. 2025;13:e20263. doi: 10.7717/peerj.20263 41180486 PMC12574594

[27] Gao X, Xu D, Li F, Baker JS, Li J, Gu Y. Biomechanical analysis of latin dancers’ lower limb during normal walking. Bioengineering (Basel). 2023;10(10):1128. doi: 10.3390/bioengineering10101128 37892858 PMC10604096

[28] Liu Y-T, Lin A-C, Chen S-F, Shih C-J, Kuo T-Y, Wang F-C, et al. Superior gait performance and balance ability in Latin dancers. Front Med (Lausanne). 2022;9:834497. doi: 10.3389/fmed.2022.834497 36091673 PMC9451043

[29] Gao X, Xu D, Baker JS, Ee-Chon T, Liang M, Gu Y. Exploring biomechanical variations in ankle joint injuries among Latin dancers with different stance patterns: Utilizing OpenSim musculoskeletal models. Front Bioeng Biotechnol. 2024;12:1359337. doi: 10.3389/fbioe.2024.1359337 38659647 PMC11039862

[30] Che L, Zhou Y, Wang Y. Effects of 12-week pilates reformer training on the biomechanics of Latin dance Cha-Cha circle chasing technique. Front Physiol. 2025;16:1549389. doi: 10.3389/fphys.2025.1549389 40534642 PMC12174401

[31] Pataky TC, Vanrenterghem J, Robinson MA, Liebl D. On the validity of statistical parametric mapping for nonuniformly and heterogeneously smooth one-dimensional biomechanical data. J Biomech. 2019;91:114–23. doi: 10.1016/j.jbiomech.2019.05.018 31155212

[32] Honert EC, Pataky TC. Timing of gait events affects whole trajectory analyses: A statistical parametric mapping sensitivity analysis of lower limb biomechanics. J Biomech. 2021;119:110329. doi: 10.1016/j.jbiomech.2021.110329 33652238

[33] Serrien B, Goossens M, Baeyens J-P. Statistical parametric mapping of biomechanical one-dimensional data with Bayesian inference. Int Biomech. 2019;6(1):9–18. doi: 10.1080/23335432.2019.1597643 34042004 PMC8211129

[34] Hartlen DC, Cronin DS. Hypothesis testing of multivariate biomechanical responses using statistical parametric mapping and arc-length re-parameterization. Ann Biomed Eng. 2025;53(10):2536–50. doi: 10.1007/s10439-025-03788-x 40659984

[35] Warmenhoven J. Over 30 years of using functional data analysis in human movement. What do we know, and is there more for sports biomechanics to learn? Sports Biomech. 2026;25(3):311–42. doi: 10.1080/14763141.2024.2398508 39475398

